# Rest-activity rhythms in small scale homelike care and traditional care for residents with dementia

**DOI:** 10.1186/s12877-017-0525-1

**Published:** 2017-07-05

**Authors:** Jeroen S. Kok, Ina J. Berg, Gerwin C. G. Blankevoort, Erik J. A. Scherder

**Affiliations:** 1 Lentis|Dignis, Mental Health Care Institute, PO Box 128, 9470 AC, Zuidlaren, The Netherlands; 2 Department of Clinical Neuropsychology, VU University Amsterdam, van der Boechorstraat 1, 1081 BT, Amsterdam, The Netherlands

**Keywords:** Dementia, Long term care, Nursing home, Actigraphy, Circadian rhythm

## Abstract

**Background:**

An enriched environment for residents with dementia may have a positive effect on the rest-activity rhythm. A small scaled homelike special care unit might be such an enriched environment. The present study shows whether the rest-activity rhythm of residents with moderate to severe dementia responds positively to a transfer from a regular Special Care Unit (SCU) to a small scaled homelike SCU.

**Methods:**

Initially, a group of 145 residents living in a regular SCU participated. Out of this group, 77 residents moved to a small scaled homelike SCU. This group was compared to the group of 68 residents that remained at the regular SCU. Rest-activity rhythm was assessed by means of actigraphy and observation scales before and after relocation.

**Results:**

No significant main effects nor significant interaction effects in intradaily and interdaily activity were found for the data of 38 residents in the small scaled homelike SCU and 20 residents of the regular SCU. The effect sizes, however, ranged from small to large.

**Conclusions:**

Considering the effect sizes, a new study with a larger number of participants is necessary before firm conclusions can be drawn.

**Trial registration:**

Current Controlled Trials ISRCTN11151241. registration date: 21–06-2017. Retrospectively registered.

## Background

Circadian rhythm disturbances, e.g. disturbances in the sleep-wake rhythm, are characteristic for aging [1], but even more so for elderly residents with dementia; circadian rhythm disturbances tend to become more severe with the progression of dementia [2–4].

A circadian rhythm is a rest-activity cycle over one day and is important for optimal functioning of an individual [5]. Observed rest-activity disturbances are long nocturnal awakenings, reduced total sleep/ sleep efficacy, restlessness [6], rapid eye movement disorders [7] and can be associated with daytime sleepiness and daytime napping [8].

Disturbances in the sleep-wake rhythm, in particular disturbed sleep during the night [9] irrespective of the type of dementia [10], is primarily a burden for the residents themselves but also for the caregivers [11, 12].

Importantly, there appears to be a close relationship between rest-activity rhythm disturbances and cognitive and behavioral dysfunctions in dementia [13, 14]. For example, residents with increased nighttime restlessness show more disturbances in executive function, memory and attention [15–18]. They also show more behavioral problems [19, 20]. Together, these clinical consequences require more intensive care [21].

It has been suggested that rest-activity rhythm disturbances might be due to a lower daytime activity level, reduced exposure to bright light, and decreased level of personal contact [22, 23]. An enriched environment can have positive effects and implies a certain level of qualitative and quantitative mental demands [24, 25], reflected in e.g. an increase in exposure to bright light during the day [13], a reduction of noise and light at nighttime [26] and specific social interventions [27] appear to improve the overall rest-activity rhythm and reduce daytime behavioral disturbances of residents with dementia [28]. Treating disturbed activity levels with psychosocial treatment, i.e. stimulation of social activities and the use of communication techniques [9, 27] and other therapies as bright light therapy at daytime [2] might relieve the work load of those who care for residents with dementia. A more stable rest-activity will also improve the quality of life of residents with dementia as the rest-activity rhythm is strongly associated with general wellbeing [29] and physical and social activity [9].

Next to a higher activity level and more bright light exposure during daytime, also other types of ‘enriched environment’ might be effective in regulating disturbances in the rest-activity rhythm of residents with dementia. One such example is a small scaled homelike Special Care Unit (SCU) for residents with dementia. These SCU’s may provide a valuable contribution to the management of rest/activity disorders in this population. It has been observed that more daily activity in SCU’s is related to a better sleep at night in residents with moderate to severe dementia [30]. Within a small scaled homelike SCU residents with dementia are encouraged to engage more in household activities like doing the laundry and cooking whereas in the more regular SCU, these services are centrally coordinated [31]. Furthermore, in small scaled homelike SCU’s (only 7 to 8 residents per unit), residents have their own private (bed)room, with a better sleeping environment. In regular SCU’s residents share their bedrooms with up to 5 residents, and live at wards with up to 20 to 30 other residents. In the small scaled homelike SCU’s, nurses are trained in so-called psychosocial treatment, i.e. they are trained in detecting problems and focus on potential causes and treatments [27, 32]. In sum, a small scaled SCU is a specific type of an ‘enriched environment’ and consequently might have a beneficial influence on the rest-activity rhythm of residents with dementia; increased activity in the form of more household activities, increased personal contact due to person centered psychosocial treatment and smaller groups [9, 23, 27] and perhaps a better sleeping environment due to single bedrooms. An increased difference between day and night, by day time interventions, can result in a stronger rest-activity rhythm due to better time cues for the resident with dementia [33]. However, a recent review shows that no previous study has investigated whether living in a small scaled SCU differs from living at a regular SCU with respect to the rest-activity rhythm of residents with dementia [34].

Therefore, the goal of the present study was to examine whether the rest-activity rhythm of residents with dementia who transferred from a regular SCU to a small scaled unit, improved in comparison to those who stayed at the regular SCU.

## Methods

### Study design

Quasi-experimental longitudinal field study with an intervention and a control group.

### Participants

Inclusion criterion was a diagnosis of dementia, exclusion criterion was no dementia.

**Fig. 1:**
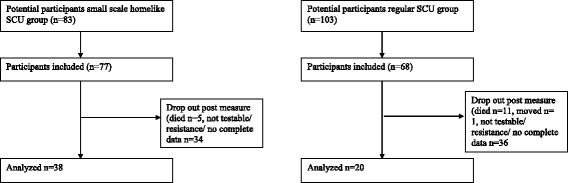
Flowchart of assessed groups, actiwatch data

The diagnosis of dementia was made by a neurologist or geriatrician (medical file) and resulted in 186 potential participants. Initially 145 participants consented: small scaled homelike SCU (*n* = 77) and control group (*n* = 68). There were no significant differences in type of dementia between both groups (see Table 1). All residents suffered from a moderate to severe dementia. For a flowchart, see Fig. 1.

**Table 1:** Type of dementia of the participants

Type dementia	Regular SCU group *N (%)*	Small scale homelike SCU group *N (%)*
Dementia nos	26 (38)	18 (23)
Alzheimer’s dementia	13 (19)	24 (31)
Vascular dementia	8 (12)	5 (7)
Mixed dementia	11 (16)	6 (8)
Frontotemporal dementia	4 (6)	0 (0)
Lewy body dementia	1 (2)	1 (1)
Other*	1 (2)	4 (5)

*Alcohol dementia, corticobasal degeneration, Korsakov, Parkinson dementia, semantic dementia

### Participant characteristics

There were no significant differences in gender, age, education, SMMSE score and depression score for both groups at the start of the study (see Table 2). To determine the global level of functioning, the Dutch version of the Standard Mini Mental State Examination (SMMSE) was used [35, 36] (19 questions with a maximum of 30 points). For depressive symptoms, the Geriatric Depression Scale (GDS) was applied [37, 38] (15 questions (yes/no)). Demographic data of the investigated subjects has been used in earlier research [39].

**Table 2:** Demographic characteristics of the participants at baseline for both groups 1 [47]

	Regular SCU group *M (SD)*	Small scale homelike SCU group *M (SD)*	Test statistic
Sample size (*n)*	48	67	
Gender	F 32, M 16	F 47, M 20	.158^a^ (n.s.)
Age (years)	82.88 (8.3)	83.27 (6.3)	.772^c^ (n.s.)
Depression	1.1 (0.9)	1.4 (1.2)	.272^c^ (n.s.)
Education^d^	3.39 (1.3)	3.32 (1.4)	.782^c^ (n.s.)
SMMSE	8.55 (6.3)	8.62 (6.5)	.961^c^ (n.s.)

^a^pearson chi square test, ^c^t-test. (two-tailed), ^d^Conform Verhage [53]

### Procedure

Initially all residents were institutionalized in two nursing homes (regular SCU’s) of a mental health care institute in the Northern part of The Netherlands. The intervention group moved from a regular SCU with wards up to 20–30 residents and bedrooms with a maximum of 5 residents to a small scaled homelike SCU. Residents in the small scaled homelike SCU had single bedrooms in separated homelike wards of 7–8 residents, situated in one large building and the nurses conceived a nine hour training focused on psychosocial treatment. The control group stayed at the regular SCU. Due to organizational reasons, one regular SCU was relocated. Rest-activity was measured with objective and subjective methods (see materials) three months before the relocation (baseline) and three months (post) and six months after relocation (follow-up).

### Informed consent

Hundred-forty-five legal representatives gave written informed consent for the study and were informed about the goal and procedure of the study. The study has been approved by the Ethical Committee of the department of Psychology of the University of Groningen, the Netherlands (no. PPO008093), registered 3 June 2009. Before each measurement the resident was asked for consent. By resistance of any kind of the resident, no measurements were conducted.

### Materials

#### Actigraphy

Objective rest-activity variables, measured by wrist movement, were assessed with an Actiwatch (Cambridge Neurotechnology Ltd., Cambridge, UK) [40], a small activity monitor with a relatively high accuracy [41]. This device contains a sensor which records intensity, amount and duration of movement in three directions. The activity of the subjects was recorded every minute, day and night, for 7 days.

The following variables were assessed: *Intra daily variability* (IV) shows the continuity or fragmentation of sleep-activity rhythm in 24 h, i.e., the extent of transitions and the frequency between rest and activity. Lower values are an indication of a normal rest-activity pattern. *Interdaily Stability* (IS) is a measure which compares all included 24 h periods from day to day (the predictability of the 24 h rest-activity pattern) and is calculated as the ratio between the variance of the average 24 h pattern around the mean and overall variance. Higher scores indicate a stable rhythm between days. *Amplitude* shows the intensity of activity or movement. A higher score represents more activity. *Relative Amplitude* (RA) serves as a measure of the relative difference (mean) in movement between the *5 least active hours* (L5) and the *10 most active hours* (M10) within an average 24 h pattern. A higher score indicates a better rhythm; a larger difference between daytime activity and night time rest. L5 represents the total activity of the 5 least active 5-h daily period (night-time rest) and M10 the total activity of the 10 most active 10-h daily period (daytime activity).

Only during showering the actiwatch was removed. A special strap prevented the subject removing the actiwatch. In case of lost periods (missing or invalid data), the actiwatch was placed for one more week. Incomplete recordings were excluded from analysis. Valid data was collected 7 × 24 h for each measurement.

The actiwatch was worn on the dominant arm during a 7-day period 3 months before the relocation (baseline), 3 months after relocation (post) and 6 months after relocation (follow-up). The control group was assessed in de same period of the year with the same time intervals.

#### Observations by nursing staff

Intersubjective activity level was assessed by using two scales of a behavioral observation scale for intramural psychogeriatry, namely restlessness and repetitive behavior (GIP) [42]. The GIP is an observation instrument which is validated to judge different behaviors of residents with dementia (4 points scale from (almost) always to never.

### Statistical analysis

The raw data collected with the actiwatches were used for analysis with SPSS, version 24. Differences between both groups were analyzed by performing independent sample t-tests and partial eta square t test (two tailed). The differences between the groups of the actigraph parameters and behavior observation scores were evaluated with a General Linear Model – multivariate variance analysis (MANOVA). *P*-values < .05 were considered as statistical significant.

Eta squared was used as measure for effect size for group mean differences [43] (95% CI) of which .01–.05 is considered as small effect size, .06–.13 as moderate and .14 and higher as large [44].

## Results

In total, complete actiwatch data were obtained from 38 participants of the intervention group and 20 residents of the control group. Measurements were done for seven days (7 × 24 h). Intersubjective measurements were obtained for 51 residents of the intervention group and 29 residents of the control group.

### Actigraphy

**Fig. 2:**
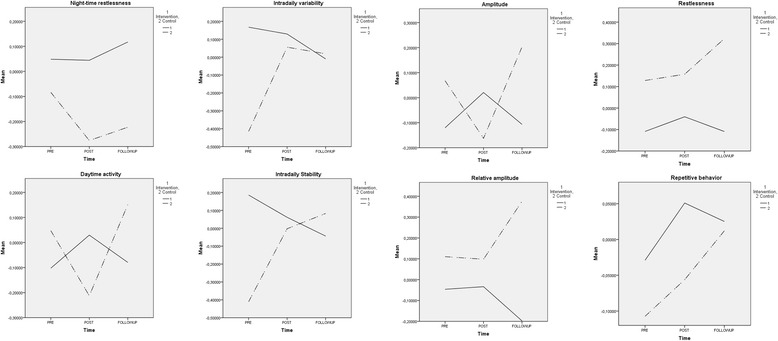
Line graphs of all variables over time (standardized)

For means and standard deviations of the actigraphic variables and interaction effects, see Table 3. Figure 2 shows line graphs for all variables.

**Table 3:** Pre-post-follow up values for both groups and differences between the groups at post test and follow-up test, controlled for pre test, interaction effects

	Experimental group (*n* = 57)			Control group (*n* = 55)						Interaction Time x group		
	Pre M (SD)	Post M (SD)^a^	Follow up M (SD)^b^	Pre M (SD)	Post M (SD)^a^	Follow up M (SD)^b^	Statistic	Value	Eta square	Statistic	Value	Eta square
Objective measures:												
Night-time restlessness (L5)	23.99 (24.71)	23.88 (27.46)	22.86 (21.30)	21.84 (20.33)	21.75 (22.92)	15.71 (20.41)	F (1406)	*P* = .241	.024	F (2672)	*P* = 0.515	.024
Daytime activity (M10)	129.29 (127.91)	120.29 (104.58)	112.71 (102.86)	132.90 (116.22)	98.70 (76.58)	142.04 (165.09)	F (1092)	*P* = .763	.002	F (2874)	*P* = .065	.095
Intradaily variability (IV)	1.26 (0.38)	1.19 (0.40)	1.24 (0.46)	1.21 (0.46)	1.24 (0.36)	1.26 (0.40)	F (1850)	*P* = .361	.015	F (2771)	*P* = .029	.121
Interdaily stability (IS)	0.39 (0.51)	0.40 (0.17)	0.33 (0.14)	0.34 (0.16)	0.37 (0.17)	0.35 (0.17)	F (1710)	*P* = .403	.013	F (2060)	*P* = .010	.155
Amplitude (AMP)	105.30 (116.70)	96.41 (88.70)	89.85 (89.67)	111.06 (109.70)	76.95 (64.65)	126.32 (158.92)	F (1333)	*P* = .566	.006	F (2804)	*P* = .069.	.093
Relative amplitude (rAMP)	0.67 (0.19)	0.69 (0.17)	0.65 (0.21)	0.69 (0.21)	0.67 (0.24)	0.77 (0.21)	F (1494)	*P* = .227	.026	F (2936)	*P* = .154	.066
Subjective measures:												
Restlessness	8.22 (2.90)	8.33 (3.15)	7.25 (2.45)	9.03 (3.82)	9.00 (3.62)	8.48 (3.46)	F (1213)	*P* = .148	.027	F (2401)	*P* = .671	.010
Repetitive behavior	8.35 (3.64)	8.49 (4.06)	7.80 (3.58)	8.07 (3.04)	8.07 (3.97)	7.76 (3.43)	F (1114)	*P* = .736	.001	F (2135)	*P* = .874	.003

^a^n = 52, ^b^n = 38

*L5* mean activity count of the 5 least active hours of the day = nocturnal activity or nighttime restlessness, *M10* mean activity count per hour of the 10 most active hours in a 24-h period = daytime activity, *IS* a periodogram-based algorithm measuring day to day stability of the rhythm, *IV* fragmentation of the activity rhythm that assesses the period to period variability of the rhythm, *AMP* amplitude, *RA* relative amplitude, (M10-L5)/(M10 + l5)

#### Main effects

There are no significant differences between both groups for the dependent variables night time restlessness, daytime activity, intradaily variability, interdaily stability, amplitude and relative amplitude (see Table 3). This implicates there is no main effect in rest-activity rhythm between residents living in a small scale homelike SCU and residents living in a regular SCU over time. Calculated effect sizes are all small; night time restlessness (.02), daytime activity (.002), intradaily variability (.02), interdaily stability (.01), amplitude (.01) and relative amplitude (.03).

#### Interaction effects

Wilks’ lambda score for interaction effects between the groups, showed two significant interactions effects (time x group) for intradaily variability and interdaily stability. The direction of the effect shows a better rest-activity pattern (intradaily stability) within 24 h for the experimental group and a more stable rhythm between days (interdaily stability) in the control group.

Four variables showed no significant interaction effects; night-time restlessness, day-time activity, amplitude and relative amplitude.

The pairwise comparisons for daytime activity and amplitude are significant for the pre – post measurements, implicating less daytime activity and a lower amplitude in the pre – post condition for both groups but not for the pre – follow up comparison. For the other variables there were no significant differences between pre – post or post – follow up.

Effect sizes (95% CI, time x group) are large for interdaily stability (.16) and moderate for daytime activity (.10), intradaily variability (.12), amplitude (.09) and relative amplitude (.07) and small for night time restlessness (.02).

### Subjective observation scores

For means and standard deviations of the observation questionnaires, see Table 3. For line graphs, see Fig. 2.

#### Main effects

There are no significant differences between both groups for restlessness and repetitive behavior implicating there were no differences in observation scores comparing the small scaled homelike SCU and the regular SCU over time. The effect sizes are small.

#### Interaction effects

For both variables, restlessness and repetitive behavior, there are no significant interaction effects between groups. The effect sizes (95% CI, time*group) for restlessness (.01) and repetitive behavior (.003) are small.

## Discussion

The present study examined whether residents with moderate to severe dementia who transferred from a regular SCU to a small scale homelike SCU would show improvements in their circadian rest-activity rhythm, measured by actigraphy and by intersubjective activity reports from caregivers. It is known that an improved rest-activity rhythm may have positive effects on quality of life for residents with dementia [2].

Our results did not show significance main effects and low effect sizes in any of the actigraphy variables. There were two significant time*group interaction effects showing a better rest-activity pattern within 24 h for the experimental group and a more stable rhythm between days for the control group. These findings are in contrast to our expectation, that was based on, among others, prior evidence that a better sleep-wake rhythm might arise from factors such as increased activity at daytime, increased personal contact and social interactions [9, 23, 27] and a quiet and dark sleeping environment [45]. One explanation of our findings compared to other studies can be a difference in used intervention. One study, for example, used a 30 min walk five times a week during 6 weeks [42] whereas in our study residents were encouraged to engage more in household activities. In the present study the residents of the intervention and control group could use comparable physical space with large walking circuits during all measures which can explain the absence of differences. So, not only the quantity of daytime activity, measured with actigraphy, but also the type of activity can account for (the lack of) outcomes. Further research is needed to evaluate the effects in quantity and quality of daytime activity for residents with dementia.

Besides this, the type of social intervention can differ in therapeutic modality; more one-to-one social interaction by family members [32] compared to, in our study, a training of the nursing personnel in psychosocial treatment. So, the type and quality of social interactions, for example (the type of) person centered care [46] or involvement by family members [47, 48] also can account for differences in care facilities. Further research exploring aspects of quality in relation to the quantity of social interactions can contribute to more specific therapeutic interventions in dementia care.

It is also possible that the relocation itself has caused the initial effect.

Effect sizes (interaction effect) vary from large to small and show mixed tendencies.

Observation scores of the nursing staff also showed no significant differences between the groups for restlessness and repetitive behavior for the residents with moderate to severe dementia. The perception by nursing staff of the rest-activity rhythm of residents with dementia can differ from the objective actigraph measures [12]. However, in this research the subjective and objective findings are in the same direction. The difference in findings between the studies can be explained by the use of different devices for the actigraphic (e.g. manufacturer or type) data and different subjective measures (type of observation measure).

The impact of environmental interventions as small scaled homelike SCU’s or light therapy on rest- activity of residents with dementia is mixed [2]. One can assume that just the differences in environment matter is too small and the measuring period too short to sort any effect on the resident with dementia [49]. A different environment can probably generate an effect in rest-activity and should perhaps be related to, for example, behavioral problems in residents with dementia [21]. Perhaps longitudinal research with repeated measurements for an extended period of time can detect the impact of environmental interventions. The question arises whether small scaled facilities attribute to better care or e.g. to the used furniture and equipment and colors in the accommodation.

### Limitations

The use of actiwatches as device in our study shows limitations such as data loss, effects due to wearing of the device and usability for residents with dementia [34]; drop out, were death, removal of actiwatches, or verbal or non-verbal resistance whereby we did not collect good contiguous periods of data [50, 51]. For a field study like this however, the maximum achievable data was collected.

Besides that, the actigraphy parameters of the used (older) actiwatches is possibly not refined enough to detect differences. Newer actiwatch versions can show more specific data and therefore more information about rest-activity rhythms of the residents with dementia [48–52]. The best practice however is the use of objective methods as well as intersubjective observational data collected from the nursing staff simultaneously [12, 40]. In dementia care, it is almost never possible to retrieve subjective information directly from the resident with dementia [34].

The current study focused on rest-activity patterns of residents with dementia and the effect of different care facilities. Further research is needed to look at other possible benefits on for example quality of life or medication use of care situations in relation to dementia.

This study is part two of a larger study performed on the same population [39]. In future articles, results about medication use, quality of life, mood and social behavior will be presented.

## Conclusion

The results show mixed differences in rest-activity for residents with moderate to severe dementia living in a small scaled homelike SCU compared with a regular SCU over time.

Longitudinal research with polysomnography and larger groups is recommended.

## Abbreviations

AMP: Amplitude
CI: Confidence interval
IS: Interdaily stability
IV: Intradaily variability
L5: Min/h activity in least active 5 h
M10: Min/h activity in most active 10 h
rAMP: relative amplitude, (M10-L5)/(M10 + l5)
SCU: Special Care Unit
